# Gut Microbiota–Metabolite–Brain Axis Reconstitution Reverses Sevoflurane-Induced Social and Synaptic Deficits in Neonatal Mice

**DOI:** 10.34133/research.0482

**Published:** 2024-09-19

**Authors:** Youyi Zhao, Sanxing Ma, Lirong Liang, Shuhui Cao, Ze Fan, Danyi He, Xiaotong Shi, Yao Zhang, Bing Liu, Meiting Zhai, Shengxi Wu, Fang Kuang, Hui Zhang

**Affiliations:** ^1^ State Key Laboratory of Oral & Maxillofacial Reconstruction and Regeneration, National Clinical Research Center for Oral Diseases, Shaanxi Engineering Research Center for Dental Materials and Advanced Manufacture, Department of Anesthesiology, School of Stomatology, Fourth Military Medical University, Xi’an, Shaanxi 710032, P. R. China.; ^2^ Department of Neurobiology and Institute of Neurosciences, School of Basic Medicine, Fourth Military Medical University, Xi’an, Shaanxi 710032, P. R. China.

## Abstract

**Background:** The mechanisms underlying social dysfunction caused by repeated sevoflurane in early life remain unclear. Whether the gut microbiota–metabolite–brain axis is involved in the mechanism of sevoflurane developmental neurotoxicity still lacks report. **Methods:** Mice received 3% sevoflurane at postnatal day (PND) 6, 7, and 8 for 2 h per day. Metagenomic sequencing and untargeted metabolomic analysis were applied to investigate the effects of sevoflurane on gut microbiota and metabolism. The animal social behavior and the synaptic development were analyzed during PND 35. Subsequently, fecal microbiota transplantation (FMT) from the control group and bile acid administration were performed to see the expected rescuing effect on socially related behaviors that were impaired by repeated sevoflurane exposure in the mice. **Results:** In the 3-chamber test, sevoflurane-exposed mice spent less time with stranger mice compared with the control group. The density of both the apical and basal spine decreased in mice exposed to sevoflurane. In addition, repeated sevoflurane exposure led to a notable alteration in the gut microbiota and metabolite synthesis, particularly bile acid. FMT reduced the production of intestinal bile acid and attenuated the effect of sevoflurane exposure on social function and synaptic development. Cholestyramine treatment mimics the protective effects of FMT. **Conclusions:** The gut microbiota–metabolite–brain axis underlies social dysfunction caused by sevoflurane exposure in early age, and bile acid regulation may be a promising intervention to this impairment.

## Introduction

Thousands of infants and children undergo general anesthesia (GA) each year for surgery or diagnostic procedures [1]*.* Evidence from numerous animal studies, including nonhuman primates, suggests that sevoflurane (Sev) exposure, especially during early neurodevelopmental stages, should have the potential to cause multiple neurodegenerative changes in the developing brain [2–4]. Additionally, clinical research has shown that repeated exposure to inhaled anesthetics in children under 3 years of age may lead to behavioral disorders and learning difficulties [5,6]. In view of the evidence presented, the Food and Drug Administration issued a drug safety communication warning that children under age 3 who are repeatedly anesthetized may suffer negative effects on their brain development [7]. Therefore, it is crucial to reveal the mechanism by which GAs affect brain development and find out effective ways to prevent potential impairments.

Neuro-apoptosis and long-term morphological/functional abnormalities in synaptic plasticity are typical pathological changes of Sev neurotoxicity and are also the main cause of subsequent neuronal and synaptic damage and eventually neurocognitive dysfunction [8–10]. Early repeated exposure to GAs has been shown to cause abnormal synaptic growth and alter cortical synaptic function in mice [6,11,12]. Aberrant synaptic occurrence and circuit formation are strongly causal with social dysfunction [11,13]. Various social behaviors are believed to be mediated by the synaptic and transmission functions of the anterior cingulate cortex (ACC) [14]. Dysfunction of excitatory synapses or inhibitory synapses in ACC may lead to social deficits in animal models [15,16]. However, increasing evidence suggests that the direct effects of Sev on neurons alone cannot fully explain the pathogenesis of Sev-induced developmental neurotoxicity [17,18]. In the past decade, treatment studies on Sev neurotoxicity, especially therapeutic strategies targeting apoptosis, neuroinflammation, and other related pathologies, have not fully explained the pathogenesis of Sev neurotoxicity [18,19]. Limitations of these studies suggest that the pathogenesis of Sev-induced neurotoxicity may attribute to systemic processes with multiple pathways or targets, rather than simply direct effects.

A correlation has been demonstrated between anesthesia exposure and gut microbiota dysbiosis [20–22]. A significant difference between the Sev exposure model and healthy controls was noted in gut microbiota composition and diversity [17,20,23]. Fecal microbiota transplantation (FMT) of the Sev-treated model to alter the composition and diversity of gut microbiota in healthy mice can lead to varying degrees of neuroinflammation and long-term cognitive impairment [17,23]. Recent reports have even shown that administration of probiotic or prebiotic is enough to mitigate the anesthesia/surgery-induced changes [24,25]. These studies emphasize that the gut microbiota intervention may be a promising therapeutic approach to prevent and treat Sev-induced neurotoxicity during development. Interestingly, gut microbiota seems to play a protective role in the prevention of neurological disorders by regulating metabolite composition [26,27]. Treatment of microbial metabolites has been shown to ameliorate behavioral abnormalities and neurological deficits in a variety of neurological disorders [28,29]. These findings indicate that further investigations are needed to elucidate the multiple effects of microbiota metabolite alteration, especially about how modifiable microbiota factors impact cognitive dysfunction after anesthesia. However, it is unclear whether the intervention of microbiota metabolites alone will affect the long-term social function of infants exposed to repeated Sev. More importantly, the effectiveness of microbial intervention or microbial metabolite interventions during critical stages of brain development in treating Sev’s neurotoxic effects is unknown.

Considering the increasing evidence of close relationship between gut microbiota and the neurotoxicity of Sev, as well as the potential regulatory effects of their metabolites on neurological function, it is meaningful to study the impact of gut microbiota and their metabolites on the neurotoxicity of Sev and how this process is mediated by the gut microbiota–metabolite–brain axis. Here we hypothesize that regulating the microbiota or microbial metabolites may be effective in alleviating neurotoxicity of neonatal Sev exposure, and found in mice that microbiota transplantation or bile acid administration significantly alleviated neuronal synaptic developmental disorder caused by repeated Sev exposure at early age, and further improved the social functional behavior in the later stage. Our work provides a new cue for understanding and a therapeutic strategy to the behavioral deficit induced by Sev exposure during early life.

## Results

### Repeated Sev exposures in early age induced social dysfunction

**Fig. 1.**
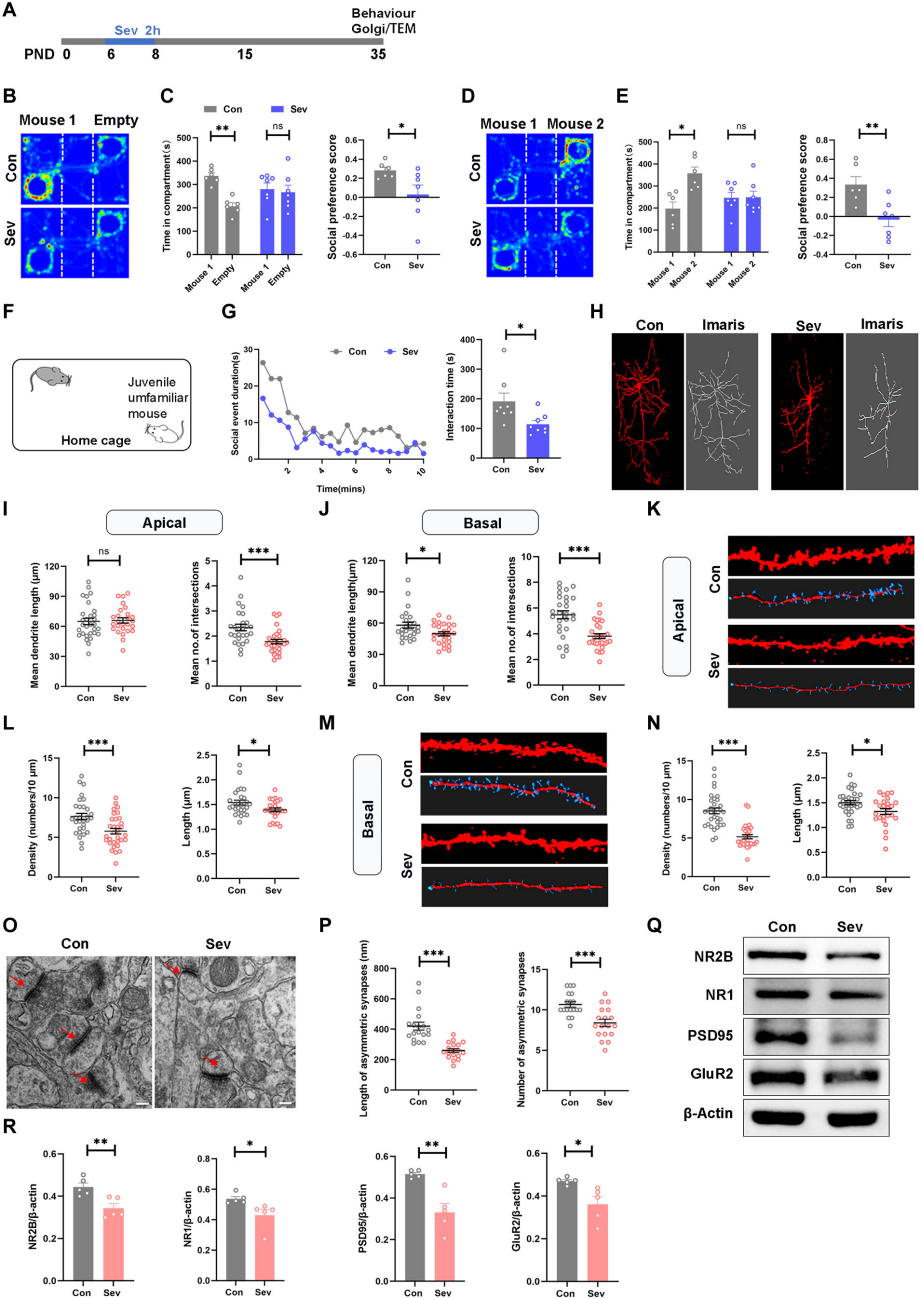
Repeated Sev exposures at early age induced social dysfunction and synaptic loss in ACC. (A) Timeline of Sev exposure and behavioral tests. (B and C) Representative heatmaps of the sociability performed in control (Con, *n* = 6) and Sev-exposed (Sev, *n* = 7) mice, and the time traveled and preference score between the mouse 1 and empty compartment. (D and E) Representative heatmaps of the social novelty of the mice, and the time traveled and preference score between the mouse 1 and mouse 2 compartment. (F and G) Diagram of residential invasion model, the contact time with invasion mice every half minute, and summary of social interaction time. (H) Typical Golgi-stained diagram and reconstructed images of representative pyramidal neurons in the ACC. Scale bars, 50 μm. (I and J) Sholl analysis of dendritic branching complexity in the apical and basal dendrites of Con and Sev mice. (K to N) Spine density and length of apical (K and L) and basal (M and N) dendrites of ACC neurons in Con and Sev mice. Scale bars, 10 μm. (O) Representative electron micrographs showing the synaptic structure on neurons (arrows). Scale bars, 100 μm. (P) Comparisons of length and number of excitatory synapses in 2 groups of mice (*n* = 18 synapses from 3 mice for each group). (Q and R) Western blotting bands and quantification for NR2B, NR1, PSD95, and GluR2 in ACC.

We determined the impact of multiple exposures to Sev in early age on long-term social behavior in mice. As shown in the schematic diagram (Fig. 1A), Sev was administered to mice 6 to 8 d after birth, followed by behavioral testing at 35 d. In the 3-chamber social experiment, mice in the Sev group spent significantly less time and distance in the social cage compared to the control group (Fig. 1B and C and Fig. S1A). The groups did not show any notable variation in movement speed (Fig. S1B). In the 3-chamber social novelty experiment, mice in the Sev group also spent significantly less time and distance in the novelty social cage compared to control mice (Fig. 1D and E). In the resident-intruder assay, mice exposed to Sev spend less time interacting with the novel mouse (Fig. 1F and G). Consistent with previous reports [17,30], we found that exposure to Sev in early life significantly impaired fear memory and increased anxiety compared to the control group of mice (Fig. S1C to E). These results indicate an impairment of early exposure to Sev on social behavior in mice.

### Repeated exposure to Sev during development led to synaptic loss and imbalanced synaptic development in ACC

The synaptic toxicity of Sev has been widely concerned. Considering the close relationship between ACC and social function, we employed Golgi staining to examine the effects of Sev exposure on synaptic development of mouse ACC 35 d afterward. Our findings indicate that Sev markedly impedes synaptic complexity and maturity during developmental stages and resulted in synaptic loss (Fig. 1H to N and Fig. S2). The electron microscopy showed aberrant synaptic structures in the ACC area of the mice exposed to Sev (Fig. 1O and P). The immunoblots also showed a significant decrease in the expression of PSD95 (a marker of postsynaptic membrane) and other synapse-related receptors in ACC of the mice after exposure to Sev (Fig. 1Q and R). These results indicate that developmental Sev exposure impairs synaptic development in ACC.

### Sev exposure altered the composition and metabolic pathway of gut microbiota in mice

**Fig. 2.**
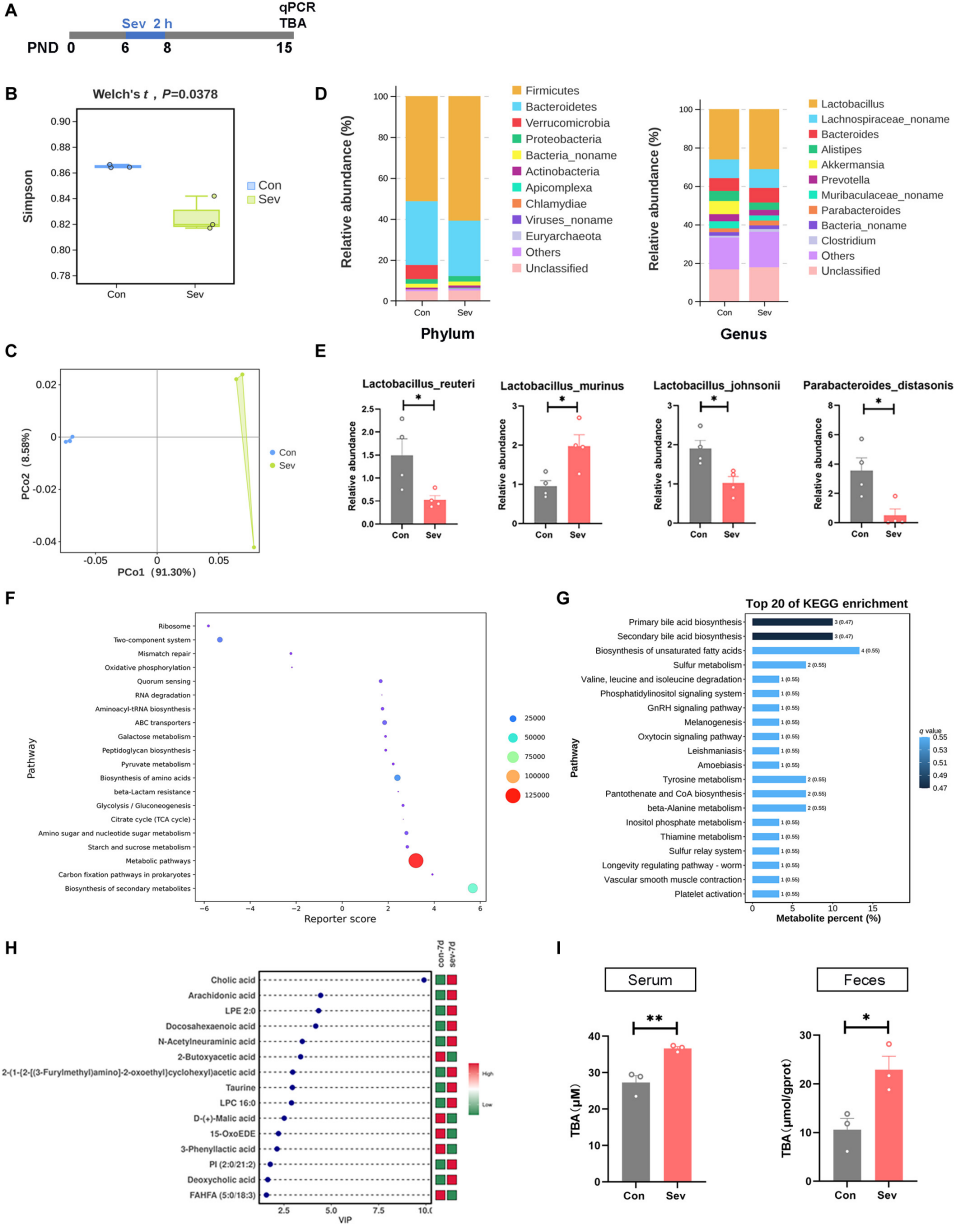
Sev exposure altered the composition and metabolic pathway of gut microbiota in mice. (A) Timeline of sequencing, qPCR, and total bile acids (TBA) tests. (B) Indices representing alpha diversity of the gut microbiota between Con and Sev groups. (C) PCoA of weighted UniFrac distance representing beta diversity of the gut microbiota in mice of both Con and Sev groups. (D) Differences in the microbiota composition at the phylum and genus levels in the Con and Sev mice. (E) qPCR validation of *Lactobacillus_reuteri*, *Lactobacillus_murinus*, *Lactobacillus_johnsonii*, and *Parabacteroides_distasonis*. (F and G) Bar plot showed the enrichment of each metabolic pathway and top 20 of metabolite enrichment. (H) Top 15 differential metabolites. (I) Detection of total bile acids in serum (left) and intestinal contents (right).

Having demonstrated the long-term social toxicity of Sev exposure during development, we investigated the potential role of the gut microbiota–metabolite–brain axis in the toxic effects of Sev on social behavior. In mice, the critical period for synaptic development spans from 1 to 3 weeks postnatally [31]. Therefore, we collected mouse fecal samples and performed metagenomic sequencing at 1 week after exposure to Sev (Fig. 2A). A statistically significant difference was found between the 2 groups when alpha diversity was quantified by Simpson (Fig. 2B). Then, the sequence data were clustered with 97% similarity as the operational taxonomic units (OTUs). Using principal coordinate analysis (PCoA) based on OTUs, distinct clustering patterns were observed among groups of samples. Interestingly, the clustering diagram of the Sev group is significantly different from the control, indicating that Sev can cause alterations in the composition of the gut microbiota (Fig. 2C). Meanwhile, Sev-treated mice showed an enrichment of *Firmicutes* and a decrease in *Bacteroidetes* in the top 10 relative abundances of key OTUs. In the terms of the abundance at the genus level, *Lactobacillus* increased, while another clade of *Bacteroides*, *Prevotella*, and *Alistipes* showed a downward trend after Sev exposure (Fig. 2D). Then, we tested the content of several strains by quantitative polymerase chain reaction (qPCR) and found that *Lactobacillus murinu* increased after repeated exposure to Sev, and *Lactobacillus reuteri, Parabacteroides distasonis,* and *Lactobacillus jonsonii* decreased, all of which were consistent with the sequencing results. Together, these results suggested that the gut microbiota of mice were dysregulated after repeated Sev exposure during development (Fig. 2E).

Next, we predicted the functional ability of Sev-induced microbial community changes. Kyoto Encyclopedia of Genes and Genomes (KEGG) pathway analysis showed that significantly different biological processes included metabolism, genetic information processing, environmental information processing, cellular processes, organismal systems, and human disease. Among them, metabolic pathways and genes were the most numerous (Fig. 2F).

To analyze metabolic changes, nontargeted metabolomics was performed with mouse Intestinal contents. KEGG analysis showed that the biosynthesis of primary bile acids and secondary bile acid was significantly enriched, including cholic acid (CA) and deoxycholic acid (DCA), in the Intestinal contents from the mice exposed to Sev at early age (Fig. 2G and H). Since our untargeted metabolomic analysis revealed that Sev substantially altered gut bile acid metabolism, we next sought to characterize the changes in Intestinal contents and serum bile acid content. Compared with control mice, the mice with Sev exposure had significantly higher levels of bile acids in both serum and Intestinal contents (Fig. 2I), suggesting that changed metabolites may due to the effect of Sev on the microbiota. These findings indicate that Sev exposure causes the gut microbiota to produce more bile acids, which seem to be absorbed and then released into the bloodstream.

### Gut microbiota transfer mitigated social deficit induced by Sev exposure

**Fig. 3.**
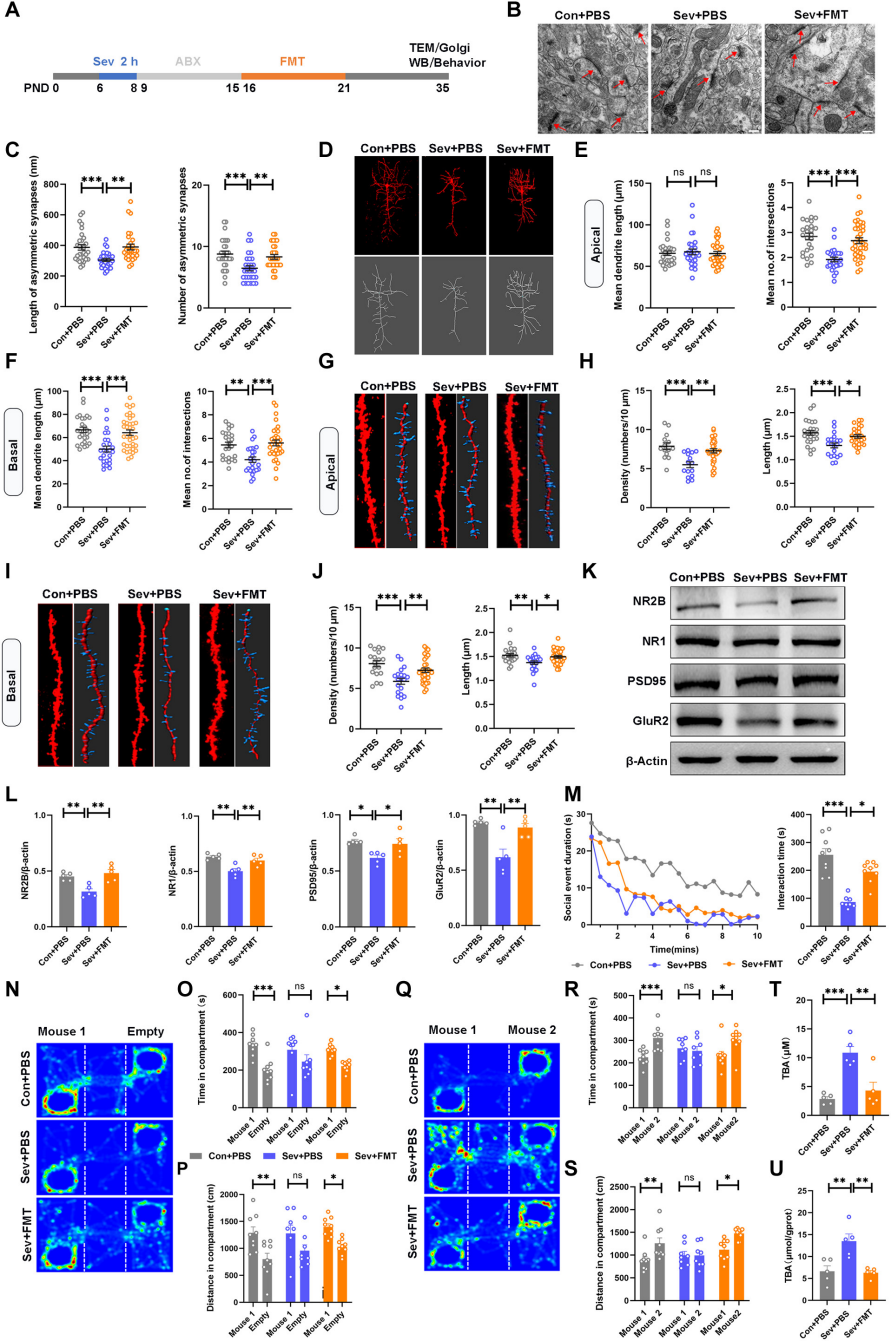
FMT mitigated social deficit and synaptic impairment induced by Sev exposure. (A) Timeline of Sev exposure, FMT, and behavioral tests. (B) Representative electron micrographs showing the synaptic structure on neurons (arrows). Scale bars, 100 nm. (C) The lengths and number of ACC asymmetric synapses in 3 groups according to electron microscopy (*n* = 29 synapses from 3 mice for each group). (D) Representative pyramidal neurons in the ACC in 3 groups of mice. Scale bars, 50 μm (E and F) Sholl analysis of dendritic branching complexity in the apical and basal dendrites based on Golgi staining. (G to J) Representative of confocal images and 3-dimensional (3D) reconstruction for pyramidal neuron dendritic spines in ACC of the mice; spine density and length of apical and basal dendrites of ACC neurons in 3 groups. (*n* = 18 to 25 spines from 3 mice for each group). Scale bars, 20 μm. (K and L) Western blotting and analyses for NR2B, NR1, PSD95, and GluR2 in ACC. (M) Summary of social interaction time spent by the 3 groups of mice with the invasion mouse. (N to P) Representative heatmaps of the sociability behavior in group of Con + PBS (*n* = 9), Sev + PBS (*n* = 8), and Sev + FMT (*n* = 9), and the time spent and distance traveled between the mouse 1 and the empty compartment. (Q to S) Representative heatmaps of the social novelty performed in Con + PBS (*n* = 9), Sev + PBS (*n* = 8), and Sev + FMT (*n* = 9) mice and the time spent and distance traveled between the mouse 1 and mouse 2 compartment. (T and U) Detection of total bile acids in serum and Intestinal contents.

Previous studies have shown a link between gut microbiota and Sev neurotoxicity [17]. To determine the influence of gut microbiota on Sev neurotoxicity, we performed FMT in young mice after Sev exposure (Fig. 3A). The results showed that the loss of synaptic number and decrease in synaptic complexity caused by Sev was significantly rescued after FMT (Sev-FMT) (Fig. 3B to F), and the reduction in spine density and length in apical and basal dendrites were also alleviated (Fig. 3G to J and Fig. S3). At the same time, the neuronal cells expressed a higher level of synapse-related proteins, including PSD95 in the group of mice that were exposed to Sev and received FMT, compared with the Sev exposure group (Fig. 3K and L). To seek out whether these microbiotas specifically reduce synaptic toxicity or improve performance on the social behavior test, we conducted social behavior tests on Sev-FMT mice. Resident-intruder assay showed that the social time of Sev-FMT mice was even very close to that of normal mice (Fig. 3M). Three-chamber tests showed that Sev-FMT mice spent more time in social chambers compared to mice that were only exposed to Sev (Fig. 3N to S). We also observed improvements in cognition and anxiety behavior in Sev-FMT mice (Fig. S4). These data demonstrated that healthy FMT alleviated synaptic toxicity and social deficits that were caused by Sev exposure at early age. Finally, the total bile acid levels in the mouse serum and feces were similar to normal ones after FMT (Fig. 3T and U). This result suggests that microbiota transplantation may regulate the metabolic pathways and the production of metabolites in the gut microbiota, and then affect the host through circulation. Due to the association between bile acids and cognitive function [32], regulating this metabolite may alleviate Sev-induced neurotoxicity.

### Cholestyramine attenuated Sev-induced social deficits and synaptic depletion

**Fig. 4.**
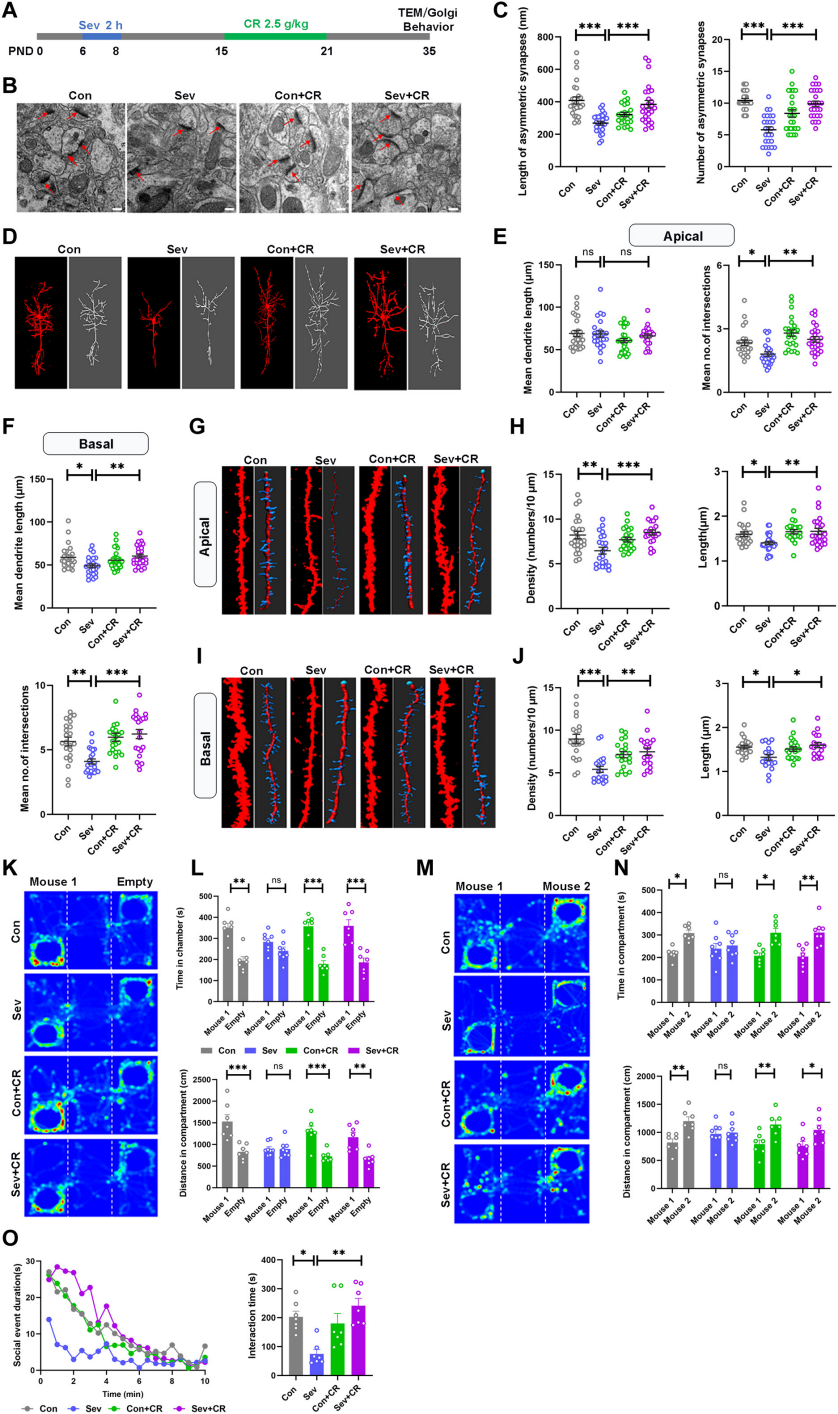
CR attenuated Sev-induced social deficits and synaptic depletion. (A) Timeline of Sev exposure, CR treatment, and behavioral tests. (B) Representative electron micrographs showing the synaptic structure (arrows) of the 4 groups. Scale bars, 100 μm. (C) Lengths and number of ACC asymmetric synapses in 4 groups according to the electron microscope (*n* = 26 synapses from 3 mice for each group). (D) Representative pyramidal neurons in the ACC in 4 groups of mice. Scale bars, 50 μm. (E and F) Sholl analysis of dendritic branching complexity in the apical and basal dendrites. (G to J) Representative reconstructed 3D confocal images of the pyramidal neuron dendritic spines in ACC of the 4 groups of mice, and analyses of spine density and length of apical and basal dendrites (*n* = 18 to 25 spines from 3 mice for each group). Scale bars, 20 μm. (K and L) Representative heatmaps of the sociability behavior in the group of Con (*n* = 7), Sev (*n* = 8), Con + CR (*n* = 7), and Sev + CR (*n* = 7) mice, and the time spent and distance traveled between the mouse 1 and empty compartment. (M and N) Representative heatmaps of the social novelty performed in the group of Con, Sev, Con + CR, and Sev + CR mice and the time spent and distance traveled between the mouse 1 and mouse 2 compartment. (O) Summary of social interaction time spent by the 4 groups of mice with the invasion mouse.

To further investigate the role of bile acids during FMT, we experimentally tested whether bile acid modulation could mimic the effect of FMT on Sev neurotoxicity. The mice were fed with cholestyramine (CR; 2.5 g/kg) from the time point at which the bile acid level increased after Sev exposure, and received behavioral tests and pathological analysis in their adulthood (Fig. 4A). According to transmission electron microscopy and Golgi staining, treatment with CR significantly increased the number and length of asymmetric synapses and increased spine density, including filopodial, long thin, stubby, and mushroom-like spines in the ACC (Fig. 4B to J and Fig. S5). Three-chamber social experiment showed that mice in the CR-Sev group spent significantly more time in the social chamber than those in the Sev group (Fig. 4K to N). In addition, CR administration significantly increased the interaction time of Sev-treated mice with stranger mice during the resident-intruder assay (Fig. 4O). Furthermore, our results showed that CR treatment could also improve anxiety behaviors without any effect on cognitive behaviors (Fig. S6A to C). Interestingly, CR did not exert a significant impact on the abundance of microbiota following exposure to Sev (Fig. S6D). These data suggest that down-regulation of intestinal bile acids attenuates Sev-induced impairment to neuronal synaptic and social functions.

### Sev down-regulated neuronal TGR5 signaling through bile acids

**Fig. 5.**
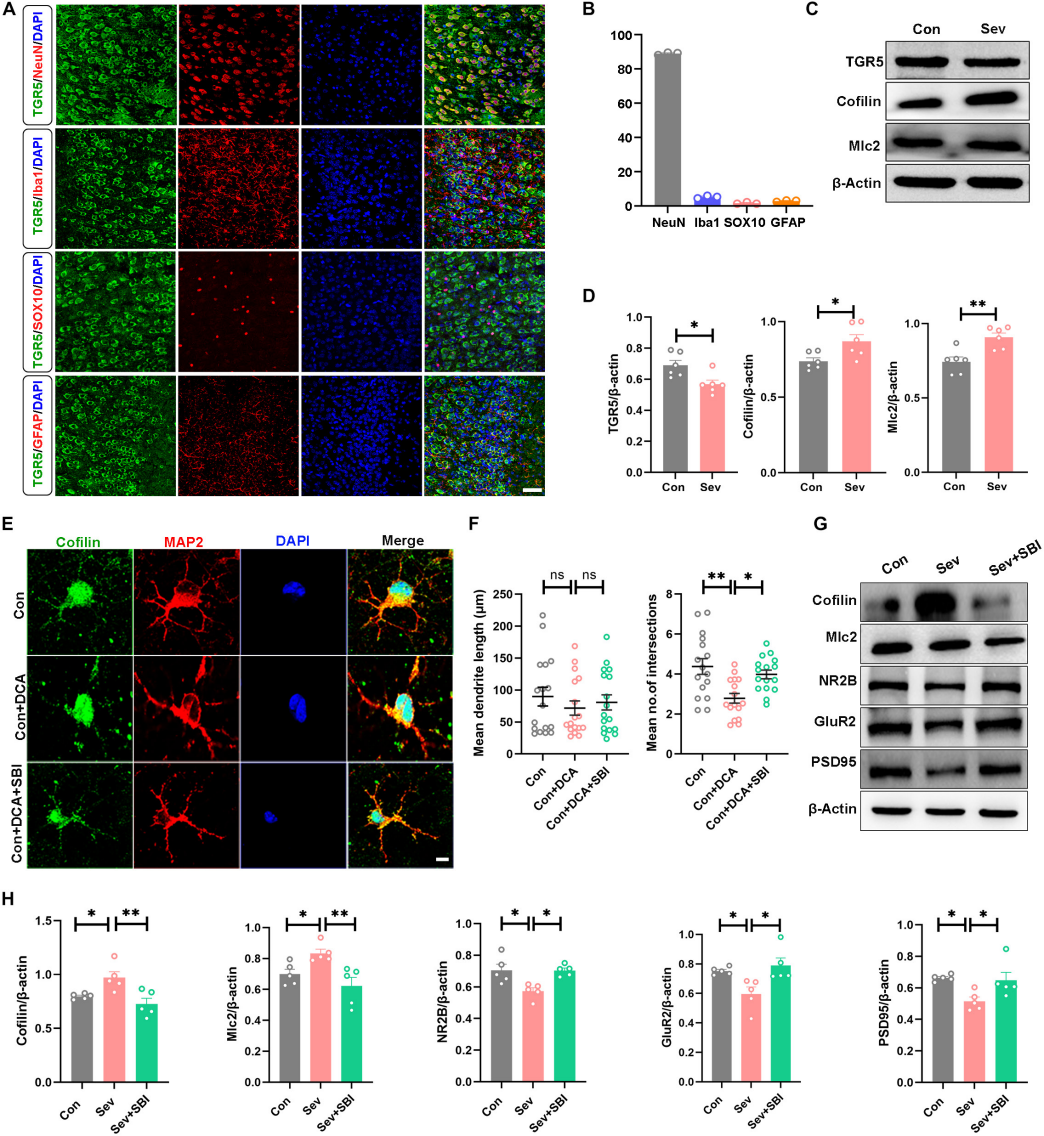
Sev down-regulated neuronal TGR5 signaling through bile acids. (A and B) Representative immunofluorescence images and quantitative summaries of TGR5 labeling in 4 cell types in ACC of the control mice. (C and D) Western blotting and analyses of TGR5, Cofilin, and Mlc2 in ACC of Con and Sev mice. (E and F) Representative immunofluorescence images of primary neurons and the complexity of the neuron dendritic analysis. (G and H) Western blotting analyses of Cofilin, Mlc2, NR2B, GluR2, and PSD95 in mice exposed to Sev and SBI-115 treatment.

G protein-coupled bile acid receptor 1 (GPBAR1 or TGR5) represents the first identified G protein-coupled receptor specific for bile acids [33]. While TGR5 is expressed in neurons, astrocytes, and microglia, its pathological and physiological roles in the brain are poorly understood [34,35]. To explore the potential mechanism of Sev damaging neuronal development through bile acid, we investigated the expression of TGR5 in conjunction with NeuN (a widely used mature neuron marker), Iba1 (a marker for microglia and macrophage), Sox10 (a marker for oligodendrocyte lineage), and GFAP (a marker for astrocyte lineage) and found that it was highly expressed in neurons in comparison to other cell types (Fig. 5A and B). Western blot showed that the TGR5 level was lowered after repeated Sev exposure, and its downstream molecules, such as cofilin and Mlc2, were significantly up-regulated (Fig. 5C and D), with cofilin being particularly associated with synaptic function [36]. Notably, our findings revealed that the alterations in several other bile acid receptors were not statistically significant (Fig. S7). To investigate the direct impact of Sev on TGR5 signaling, we exposed primary neurons to Sev and subsequently assessed TGR5 expression via Western blot analysis. Our findings indicate that direct Sev exposure does not significantly alter TGR5 expression in neurons (Fig. S8A). Consequently, we propose that alterations in gut microbiota and its metabolites are critical factors contributing to the observed changes in neuronal TGR5 following Sev exposure. Previous studies have suggested a correlation between alterations in TGR5 and inflammatory processes [37]. So we investigated whether neuronal inflammatory factors are changed following Sev exposure. Western blot analysis revealed that, consistent with prior findings [38], the levels of inflammation-associated cytokines tumor necrosis factor-α (TNF-α) and interleukin-6 (IL-6) and the transcription factor nuclear factor κB (NF-κB) in primary neurons did not exhibit significant changes after Sev exposure (Fig. S8B). These results indicate that Sev does not influence neural function via the inflammation-related signaling pathway of TGR5. Next, we investigated the effect of the TGR5 inhibitor SBI-115 on the neurotoxicity of Sev. The administration of SBI-115 resulted in the suppression of TGR5 expression levels; however, it did not influence neuronal synaptic development (Fig. S9). Three days of DCA treatment mimicked the synaptic effects of repeated exposure to Sev during development, and supplementation with SBI-115 (10 μM) can significantly mitigate the DCA-induced reduction in neuronal dendrite complexity (Fig. 5E and F). Administration of SBI-115 (80 mg/kg) almost restored synapse development in the ACC brain region, indicated by Western blot (Fig. 5G and H). The above findings suggest that Sev down-regulates the expression of the TGR5 signaling pathway via bile acids. Furthermore, modulation of TGR5 expression may mitigate the synaptic toxicity induced by Sev.

## Discussion

**Fig. 6.**
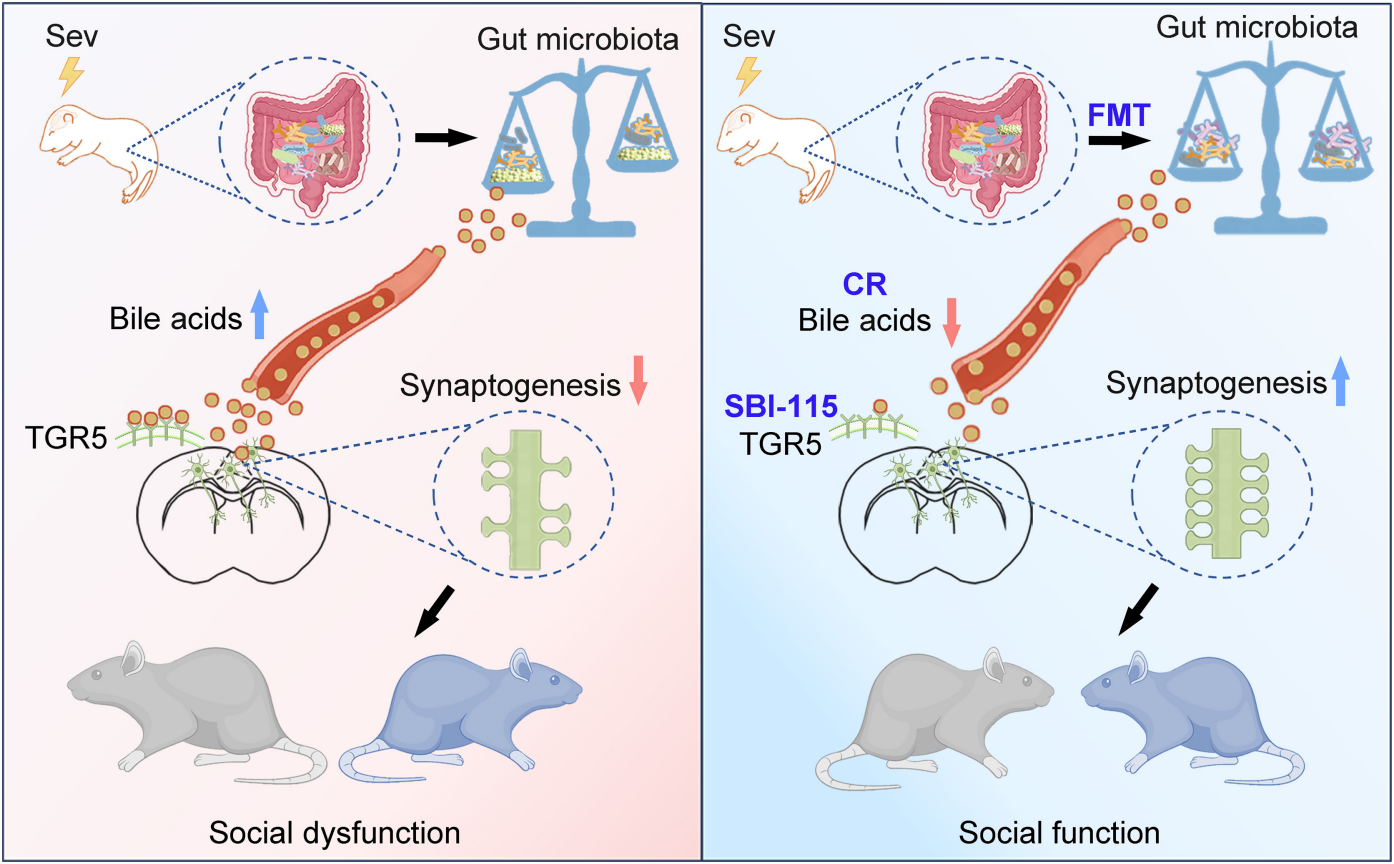
Graphical abstract. The microbiota–metabolite–brain axis underlies social dysfunction caused by Sev, and bile acid regulation may be a promising intervention.

There is growing evidence that neonatal Sev exposure has lasting neurotoxic effects in adulthood, including cognitive dysfunction, social dysfunction, and mood abnormalities [19,39,40]. Nevertheless, the specific mechanism by which Sev causes neurotoxicity remains unclear [19,41]. In this study, we found that the gut microbiota–metabolite–brain axis was involved in the toxic effects of Sev on social behavior and synapse development. Importantly, we showed that FMT prevents the long-term toxic effects induced by Sev and that modulation of metabolites, particularly bile acids, mimics this therapeutic effect. Our research therefore highlights the role of the gut microbiota–metabolite–brain axis in Sev-mediated neurotoxicity during the neonatal period and suggests that remodeling this axis should be a potential therapeutic strategy for preventing neurotoxicity produced by Sev exposure during development (Fig. 6).

Known as the gut microbiota–brain axis, it refers to an information network linking the gut microbiota with the brain and is critical to maintaining body and nervous system function and homeostasis [42]. There is a lot of evidence that imbalances in the gut microbiota are related to the pathogenesis of several nervous system abnormalities [43,44], including anesthetic neurotoxicity [45]. It was reported that the gut microbiota was disrupted after GA exposure [22,46], and FMT experiments revealed a relationship between gut microbiota and Sev exposure-induced changes in behavior [17]. Similarly to previous studies, our study found that Sev exposure affected gut microbiota composition significantly. Notably, FMT was sufficient to block the toxic effects of Sev exposure. This suggests that the neurotoxic effects induced by Sev may be related to the function of microorganisms. In particular, Sev exposure can significantly reduce the abundance of *Bacteroidetes*, which was associated with the accumulation of DCA that has been shown to be involved in the pathogenesis of various neurological disorders [32,47–49]. Furthermore, our results show that FMT of healthy animals can significantly reduce Sev-induced synaptic and social toxicity, as demonstrated by the recovery of dendritic spine development and the three-chamber test. We believe that changes in the microbiome, and microbiome-derived metabolites, specifically, are driving the social behavioral dysfunction. We did not investigate whether anesthesia-related neurotoxicity depends on the microbiome, as previous reports have shown that the absence of microorganisms has a serious impact on social interaction [50]. Further research is needed in the future to show microbiome dependence of anesthesia-induced neurotoxicity. The precise concentration of Sev in the digestive tract of juvenile mice following exposure remains undetermined, complicating the investigation of its direct effects on gut microbiota. Although anesthetics may exert direct effects on the gut microbiota via systemic circulation, the resultant alterations in microbial composition are not uniformly observed across different studies [17,51]. It is posited that the systemic effects of Sev on mice, including changes in metabolism, intestinal peristalsis, and feeding behavior, may play roles in its impact on gut microbiota. Meanwhile, this study primarily focused on the effect of microbiota transplantation on Sev neurotoxicity. Future mechanistic studies should aim to investigate the therapeutic effect of supplementation with specific probiotics on Sev-induced neurotoxicity.

There is a very complex relationship between gut microbiota and host metabolism [52]. Gut microbiota-derived metabolites, such as short-chain fatty acids, tryptophan metabolites, D-amino acids, and bile acids, may play a crucial role in the pathophysiological condition of neurological disorders via the gut microbiota–brain axis [53,54]. It can therefore be speculated that the change of the gut microbiota through Sev exposure will lead to metabolic alterations. Indeed, we found clear effects of Sev on gut microbes and their metabolic characteristics based on our functional analysis of gut microbes and metabolic analysis of cecal contents. These changes included a significant increase in bile acid biosynthesis (both primary and secondary bile acid) and a decrease in short-chain fatty acid (for example, levulinic acid and 2-butoxyacetic acid). In particular, bile acid metabolism has been found to be associated with the pathogenesis of autism [55,56]. Interestingly, CR has been shown to have therapeutic effects on multiple mouse models of neurological pathology [57,58], and in our study, the negative effects of Sev on synaptic development were attenuated by CR treatment. Thus, the neurotoxic effects of Sev may be due to its combined actions on the gut microbiota and its metabolites. In addition, CA or DCA feeding aggravated neurological decline in neuropathological model [59], suggesting that gut-associated bile acids may influence neurological development, although precisely how they dysregulate the development of neural cells and synapses is unclear yet. While our findings indicate that gut microbiota bile acids are important contributors in Sev-induced social dysfunction, other related pathways in the gut–brain axis may also contribute to the neurotoxicity of Sev. Other metabolites, such as levulinic acid and phenyllactic acid [60], also need further investigation in the neurotoxic effects of Sev. In short, these changes in gut microbial metabolism partially explain the mechanism by which Sev causes neurotoxic effects. These results highlight the critical role of the gut microbiota–metabolite–brain axis in the mechanism of Sev-induced long-term neurotoxic effects.

Another important finding is that the negative effects of Sev or DCA were blocked in vivo and in vitro experiments with the TGR5 blocker (SBI-115). We found that TGR5 blocker SBI-115 administration helped attenuate the neurotoxicity induced by Sev exposure. This is striking because it demonstrates a possible mechanism by which abnormal bile acid metabolism affects neuronal function and provides a future target for the treatment to Sev-induced neurotoxic effects. Meanwhile, TGR5 and downstream pathways were closely associated with neural function. For example, TGR5-specific agonist (INT-777) administration significantly reduced activation of microglial cells, brain edema, and neuroinflammation in neuropathological models [61]. On the other hand, intracerebroventricular administration of INT-777 produced anorexic effects in wild-type mice [62], suggesting that TGR5 may be controversial due to its complex and multifaceted roles in the nervous system. Interestingly, our findings showed that TGR5 inhibition with SBI-115 may protect against long-term synaptic developmental impairment caused by abnormal increase of bile acids after Sev exposure. Concurrently, the application of SBI-115 demonstrates the therapeutic potential of modulating TGR5 in clinical patients. The beneficial effect of TGR5 inhibition on the neurotoxicity of Sev again supports the idea that the gut microbiota–metabolite–brain axis is involved in Sev-induced neurotoxicity. Thus, our findings provide a therapeutic strategy for the prevention of Sev neurotoxicity by reshaping the gut microbiota–metabolite–brain axis. We further validated the pertinent pathways and observed that a reduction in TGR5 expression influenced the expression of cofilin, a protein whose stability is essential for the maintenance of synaptic plasticity. This finding suggests that TGR5 may impact synaptic development in an indirect way.

Although our work and recent researches have highlighted the neuroprotective effects of microbiota and their metabolites, there are different reports suggesting that bile acid regulation may be harmful to the nervous system [62]. Hence, further basic and clinical researches are needed before applying microbial-metabolite modulation to prevent or treat Sev neurotoxicity.

## Methods

### Animals

C57BL/6J background wild-type mice were obtained from the Fourth Military Medical University’s experimental center. A constant temperature and 12-h/12-h light/dark cycle were maintained for all mice. Water and food are freely available to mice. All mice were randomly assigned to receive the experimental treatment within the same group for the experiment. Light cycle was used to conduct all experiments.

### General anesthesia

Six-day-old mouse pups were randomly allocated to either the control group or the anesthesia group. Mice assigned to the anesthesia group were placed in an anesthesia chamber and subjected to Sev anesthesia, receiving 3% Sev for a duration of 2 h daily over the course of 3 consecutive days. Throughout the anesthesia procedure, a constant temperature was maintained, and 60% oxygen was administered at a consistent flow rate. Following the anesthesia sessions, the mice were returned to their mothers and continued to be nursed. The control group mice underwent identical handling procedures, with the exception that they were not exposed to Sev. For primary neurons, Sev was transported from the anesthesia machine to a sealed plastic box maintained at 37 °C in an incubator. On the third day in vitro, the neurons were exposed to a gas mixture containing 3% Sev (21% oxygen, 5% carbon dioxide, and 3% Sev) for a duration of 2 h per day over a period of 3 d. Neurons were subsequently collected for experimental analysis after 14 d in vitro.

### CR treatment

The mice of the control and Sev-treated groups were given oral CR at a dose of 2.5 g/kg [57,63] from postnatal day (PND) 15 onward for 7 consecutive days, and the social function and synaptic development of the mice were detected at PND 35.

### Antibiotic treatment and FMT

In the aftermath of Sev treatment, antibiotics were given to deplete the gut microbiota, according to the study [17]. A daily antibiotic mix of 0.1 ml (vancomycin 0.5 g/l, imipenem 0.25 g/l, ciprofloxacin 0.02 g/l, metronidazole 1 g/l, ampicillin 1 g/l) was administered by gavage at PND 9 to PND 14 once every other day. FMT started from PND 15: Fecal samples of the control mice at PND 15 were taken, homogenized in phosphate-buffered saline (PBS) (1 ml/0.1 g Intestinal contents), and centrifugated, and then the supernatant was administered orally every day for a week as 0.1 ml. Social function and synaptic development were estimated at PND 35.

### Golgi staining and spine analysis

Isoflurane was used to anesthetize mice, and PBS (pH 7.4) was infused through the heart. A mixture of 50% potassium bichromate, 5% mercury chloride, and 5% potassium chromate was used to stain brain tissues for a week in the dark. Using distilled water, ethanol, and ammonia (3:1), 150-μm brain sections were cleaned and dehydrated. Incubation in 5% sodium thiosulfate for 10 min was followed by dehydration with degraded ethanol and clarification with xylene. An examination of dendrite branching was conducted using sholl analysis. In the ACC region, IMARIS was used to reconstruct and analyze dendrite spine density and length of pyramidal neurons.

### Transmission electron microscopy

Isoflurane was used to anesthetize mice. PBS (pH 7.4), 2% polyformaldehyde phosphate buffer, and 2% glutaraldehyde mixture were infused through the heart. The brains tissue was taken and postfixed for 3 h. Then, 50-μm ACC coronal sections were cut by vibratome (VT1000S, Leica), dehydrated in gradient ethanol and propylene oxide, and flat-embedded in Epon 812 (SPI-Chem) after 1.5 h in 1% osmium tetroxide in 0.1 M phosphate buffer. JEM-1230 electron microscope (JEOL Ltd.) was used to observe ultrathin sections cut with an ultramicrotome (EMUC6, Leica), mounted on grides, and stained with uranyl acetate and lead citrate. Gatan digital camera and its application software (832 SC1000) were used to take electron micrographs. The lengths of postsynaptic terminals were measured with ImageJ-Pro Plus 6.0.

### Immunohistochemistry

Isoflurane was used to anesthetize mice, and PBS (pH7.4) and 4% polyformaldehyde phosphate buffer were infused through the heart. The brains were removed and fixed at 4 °C with the same fixative for 4 h. After being immersed overnight with 20% sucrose and 30% sucrose successively, cryostats were used to slice the brains into 25-μm-thick slices. Slices of brain were incubated overnight with rabbit anti-TGR5 (ABclonal, A20778, 1:200), mouse anti-NeuN (Abcam, ab104224, 1: 600, RRID: AB_10711040), chicken anti-GFAP (Genetex, GTX85454, 1:200, RRID: AB_10621124), mouse anti-SOX10 (Oasis Biofarm, OB-PGP001, 1:500, RRID: AB_2934230), and goat anti-Iba1 (Wako, 019-19741, 1:200, RRID: AB_839504) in PBS containing 3% bovine serum albumin and 0.3% Triton-X100. Incubation was conducted at room temperature for 2 h away from light with corresponding secondary antibodies conjugated with Alexa Fluro 488 (Donkey Anti-Rabbit, Jackson Institute for Immunology, Jackson 1:500, RRID: AB_2313584), Alexa Fluro 594 (Donkey Anti-Mouse, Jackson Institute for Immunology, Jackson 1:500, RRID: AB_2338372), or Alexa Fluro 594 (Donkey Anti-Chicken, Jackson Institute for Immunology, Jackson 1:500, RRID: AB_2340377). 4′,6-Diamidino-2-phenylindole (1:1,000, Sigma) was used to stain slices 30 min after washing with PBS. Finally, the sections were covered with a mixture of 50% glycerol and 50% PBS.

### Three-chamber test

Behavior arena dimensions were 65 × 45 × 25 cm^3^ with 3 chambers following the same pattern, each of which had a size of 43 × 23 cm^2^ (internal area of the chamber). The sociability test was conducted by placing a mouse in the middle chamber for 10 min and then inserting a cylinder containing a novel mouse into the left chamber and the empty cylinder into the right chamber. It took 10 min for the second social unfamiliar mouse in the right chamber to complete the social novelty test. The subject mouse was measured for the amount of time and distance spent in each compartment. A 75% ethanol solution was used to clean each chamber prior to testing to minimize odor influence. The behavior was analyzed using SMART 3.0 software (Panlab Harvard Apparatus).

### Social interaction in the home cage

Before the test, each experimental mouse should be kept in a single cage for 24 h, and a 5-week-old mouse was introduced into the cage and the subject mouse was allowed to explore it freely for 10 min afterward. A number of social interaction behaviors were observed, including close following, physical sniffing initiated, and direct contact.

### Western blotting

The radioimmunoprecipitation assay buffer containing proteinase inhibitor cocktail was used to lyse ACC tissues. The bicinchoninic acid (BCA) assay was used to quantify the protein samples, sodium dodecyl sulfate–polyacrylamide gel electrophoresis was used to resolve them, and polyvinylidene difluoride membranes were used for transfer. Following blocking with tris-buffered saline containing 5% nonfat milk and 0.1% Tween 20, primary antibodies were incubated overnight at 4 °C as follows: mouse anti-actin (Proteintech, 81115-1-RR, 1:5,000, RRID: AB_2923704), rabbit anti-GluR1 (Abcam, ab109450, 1:1,000, RRID: AB_10860361), rabbit anti-GluR2 (Abcam, ab133477, 1:1,000, RRID: AB_2620181), rabbit anti-PSD95 (Abcam, ab18258, 1:1,000, RRID: AB_444362), rabbit anti-synaptophysin (Abcam, ab32127,1:20,000, RRID: AB_2286949), rabbit anti-NR2A (Abcam, ab106590, 1:1,000, RRID: AB_10861125), rabbit anti-NR2B (Abcam, ab183942, 1:1,000, RRID: AB_2889878), and rabbit anti-NR1 (Abcam, ab17345, 1:1,000, RRID: AB_776808). A Thermo ECL kit was used to visualize bands on the membranes after incubation with secondary antibodies conjugated with horseradish peroxidase. Images were quantified with ImageJ by determining the ratio of gray scales of the target protein and β-actin after the blot images were acquired with a Tanon imaging system.

### Metagenomic sequencing and analysis

Fecal samples were collected from mice in the control and Sev groups, and genomic DNA was extracted from the samples according to the instructions of the HiPure bacterial DNA kit and sequenced on an Illumina Novaseq 6000 sequencer. DNA fragments of 300 to 400 base pairs were amplified by PCR and subsequently using an AMPure XP system (Beckman Coulter, Brea, CA, USA). Purified PCR products with Agilent 2100 bioanalyzer (Agilent, Santa Clara, CA) were used to examine the sequenced libraries, and reverse transcription PCR (RT-PCR) was used for library quantification.

To assess bacterial richness, alpha diversity (Simpson index used in this study) and beta diversity differences (PCoA used in this study) were assessed using the Vegan package in R (version 2.5.3). Cluster analysis of species composition was carried out.

### Nontargeted metabolomic analysis

Fecal samples of mice in the control and Sev groups were collected for nontargeted metabolic analysis. The samples were separated by Agilent 1290 Infinity LC ultrahigh performance liquid chromatography (UHPLC) HILIC column. AB Triple TOF 6600 mass spectrometer was used to collect the primary and secondary spectra of the samples. All metabolites were enriched by the metabolic pathway of KEGG differential metabolites using R-package pheatmap (v1.0.12). The abundance of differential metabolites in the same group was normalized by z-score, combined with VIP (variable) of OPLS-DA. The Importance in projection value is plotted to compare the top 15 differential metabolites with the largest VIP value in the group.

### DNA isolation and real-time qPCR analysis

Bacterial DNA was extracted from mouse Intestinal contents using TIANamp DNA fecal kit. The qPCR mixture contained 5 ng of tissue DNA and 1 μl of upstream primer and downstream primer and was configured with water to form a 20-μl system. All bacteria (forward: 5′-TGSTGCAYGGYTGTCGTCA-3′ and reverse: 5′-ACGTCRTCCMCACCTTCCTC-3′); *Lactobacillus murinu* (forward: 5′-TCGAACGAAACTTCTTTATCACC-3′ and reverse: 5′-CGTTCGCCACTCAACTCTTT-3′); *Parabacteroides distasonis* (forward: 5′-GGACACGTCCCGCACTTTAT-3′ and reverse: 5′-TTCTGAGAGGAAGGTCCCCC-3′); *Lactobacillus reuteri* (forward: 5′-GAAGATCAGTCGCAYTGGCCCAA-3′ and reverse: 5′-TCCATTGTGGCCGATCAG-3′); *Lactobacillus jonsonii* (forward: 5′-TCGAGCGAGCTTGCCTAGATGA-3′ and reverse: 5′-TCCGGACAACGCTTGCCACC-3′)were used as the internal parameter. PCR amplification was performed using the following cyclic parameters: 1 min at 95 °C; 39 cycles of 10 s at 95 °C, 30 s at 60 °C, and 30 s at 70 °C. A comparison of different experimental groups was conducted using the ΔΔCt method.

### Bile acid assessments

The total bile acid kit (E-BC-K181-M) was used to detect changes in stool and serum bile acid content, and the procedure was carried out according to the instructions. Serum was directly measured at 405 nm, while Intestinal contents were homogenized with normal saline and the supernatant was taken. Ten microliters of serum and fecal supernatant was taken, 200 μl of A solution was added, and then 50 μl of B solution was added and mixed well. It was put in 37 °C oven for 3 min, and the absorbance was measured at 405 nm, as A1. Then, it was put in 37 °C oven for 5 min, the absorbance was measured, as A2, and the bile acid content of the sample was calculated.

### Primary neuron culture

The E14-16 mice were obtained and placed in sterilized culture dishes. The whole brain was extracted and placed in a culture dish containing precooled PBS. The cortical tissue was isolated, and the meninges were removed and cut into small pieces. Trypsin (0.25%) was added and incubated at 37 °C for 15 min to facilitate digestion. Enzyme digestion was terminated by adding Dulbecco’s modified Eagle’s medium, and repeated pipetting was performed to dissociate the cells. The supernatant was collected and centrifuged to pellet the cells. The cells were resuspended in serum-free medium (98% neurobasal medium, 1% b27 supplement, and 1% glutamine). The cell density was adjusted, and the cells were seeded onto a cell culture plate. The cells were continuously cultured in the incubator, and the culture medium was replaced every 48 h subsequently.

### Statistical analysis and figure preparation

Means and standard errors are reported as mean ± SEM. GraphPad Prism 9 was used to analyze the data. A minimum of 6 mice were allocated to each group for the behavioral assay. Normality tests and homogeneity of variance tests were carried out using Shapiro–Wilk and Levene’s tests, respectively. The following analyses were performed for data that conformed to normality and homogeneity of variance: unpaired 2-tailed *t* tests, Tukey’s one-factor variance tests for multiple comparisons, and repeated-measures analysis of variance. In the case of data not conforming, the Mann–Whitney *U* test or Kruskal Wallis test and Dunn multiple comparison test, Welch *t* test, or Wilkerson signed rank test was employed. *P* < 0.05 was considered statistically significant. Adobe Photoshop was used to assemble the figures. Some cartoon components were provided by Figdraw.

## Data Availability

The datasets used and/or analyzed during the current study are available from the corresponding author on reasonable request.
