# Supplementary figures and images for: Gut Microbiota–Metabolite–Brain Axis Reconstitution Reverses Sevoflurane-Induced Social and Synaptic Deficits in Neonatal Mice
Source: Research (Wash D C). 2024 Sep 19;7:0482. doi: 10.34133/research.0482 (PMC11411162; doi:10.34133/research.0482)

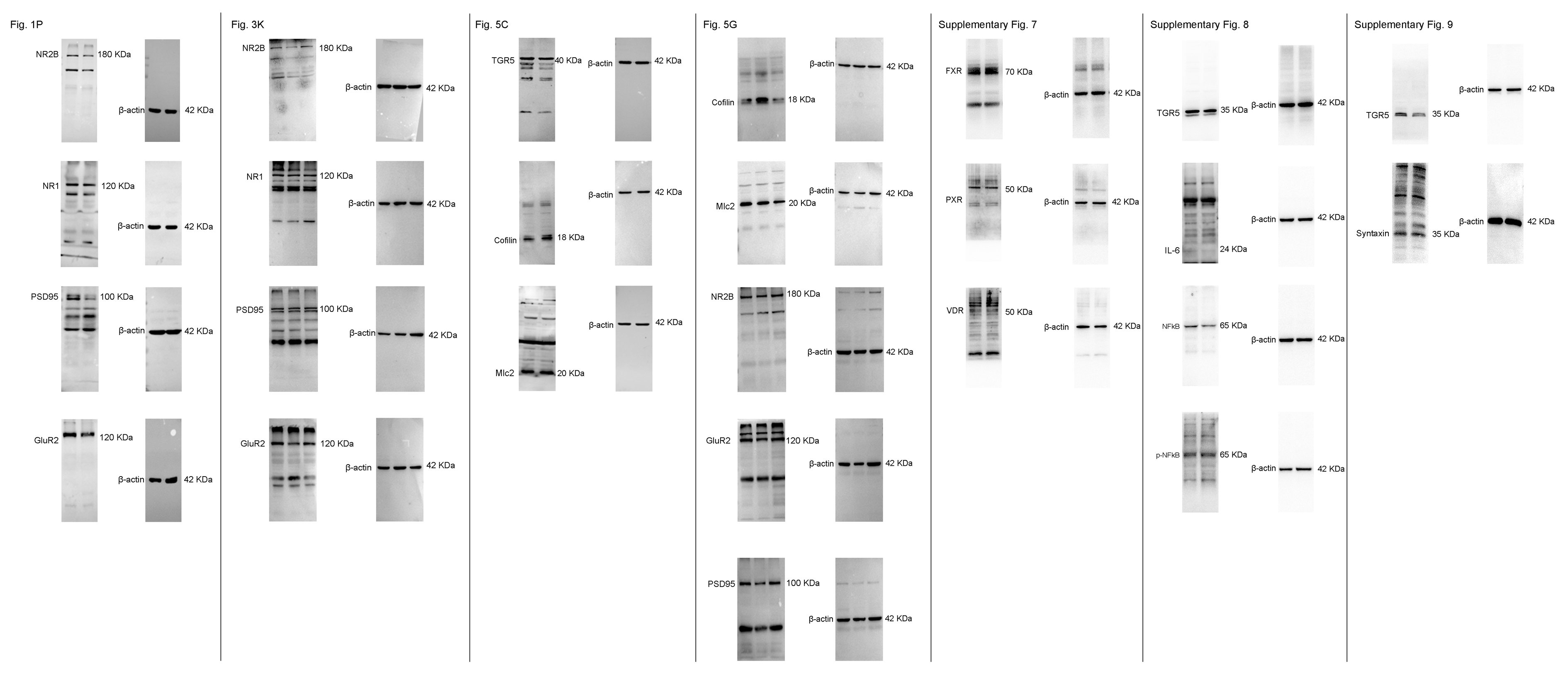

Supplement: Supplementary 1 — Figs. S1 to S9 [file research.0482.f1.zip › Full gel images of figures.tif]

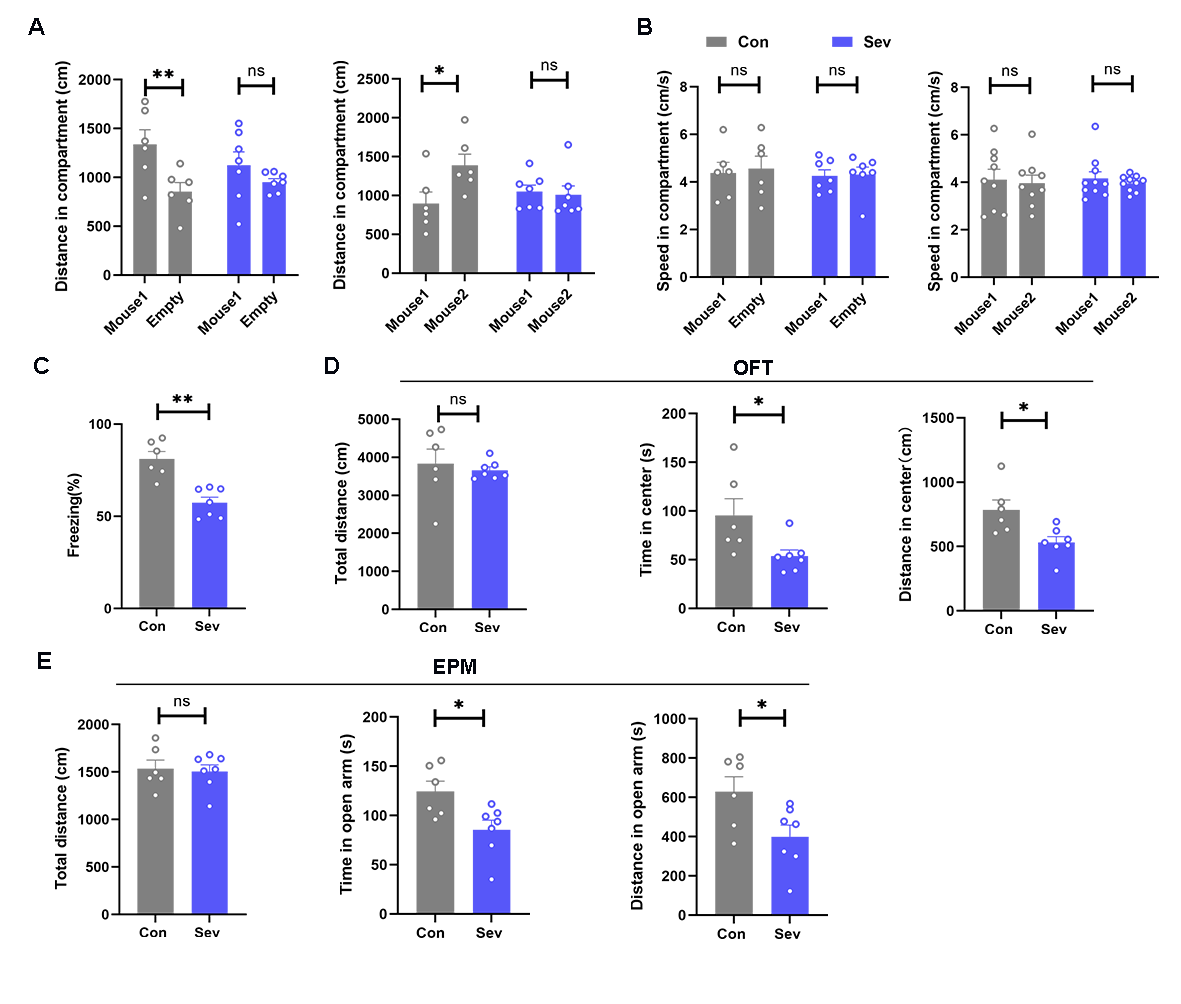

Supplement: Supplementary 1 — Figs. S1 to S9 [file research.0482.f1.zip › Supplementary Figure 1.tif]

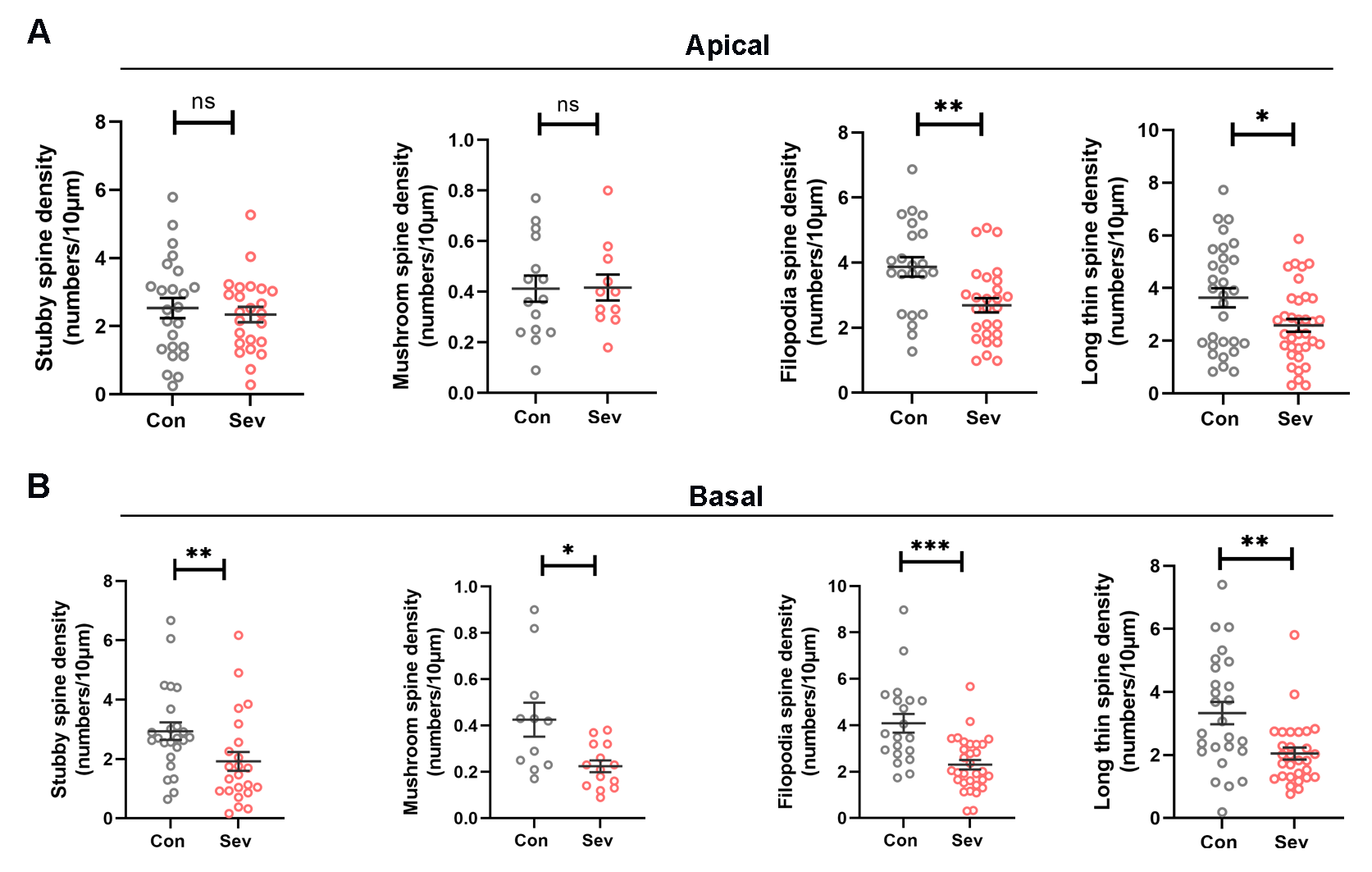

Supplement: Supplementary 1 — Figs. S1 to S9 [file research.0482.f1.zip › Supplementary Figure 2.tif]

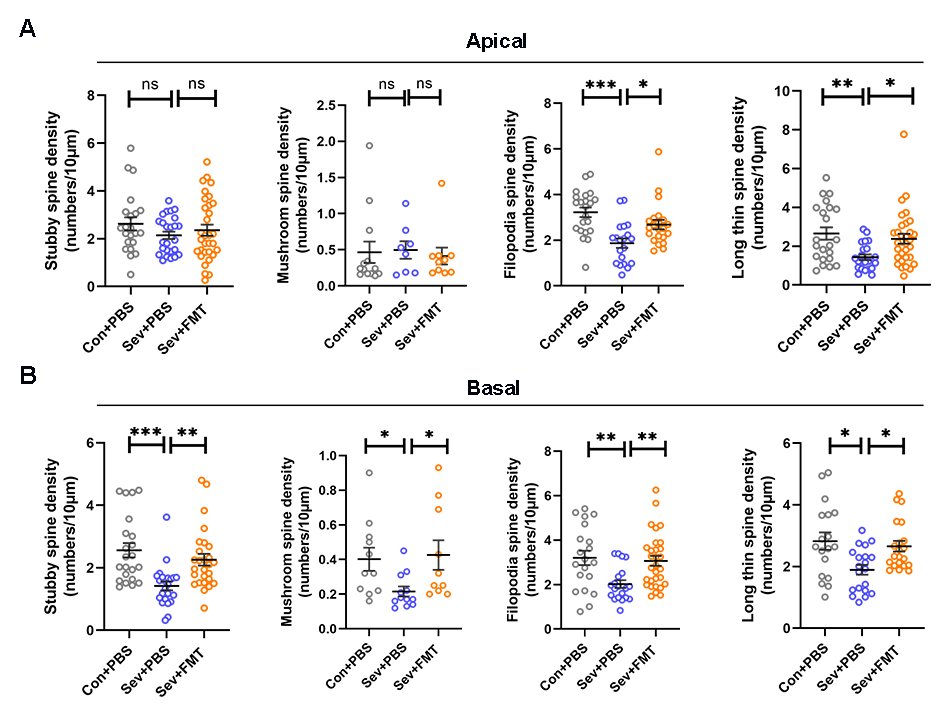

Supplement: Supplementary 1 — Figs. S1 to S9 [file research.0482.f1.zip › Supplementary Figure 3.tif]

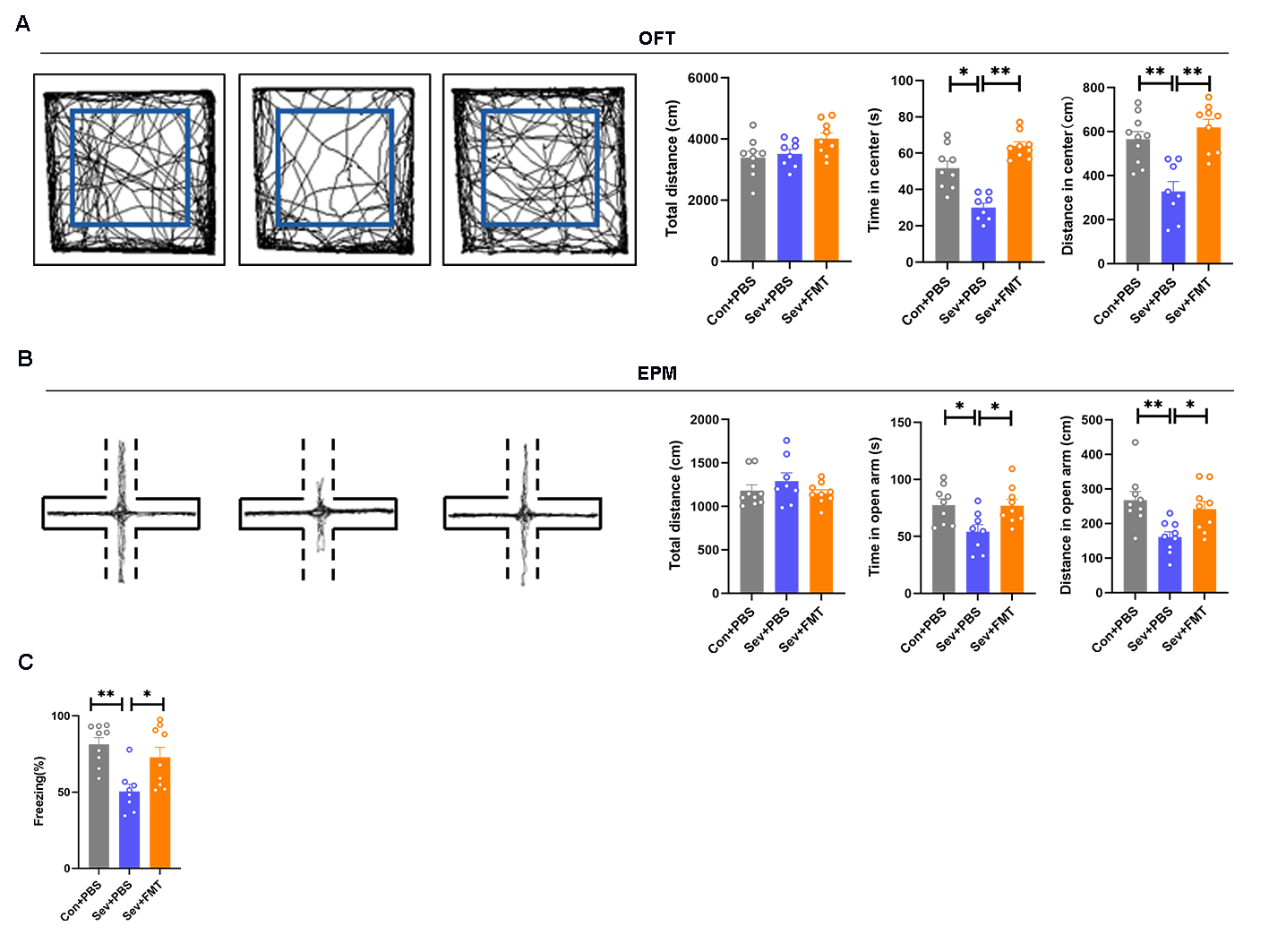

Supplement: Supplementary 1 — Figs. S1 to S9 [file research.0482.f1.zip › Supplementary Figure 4.tif]

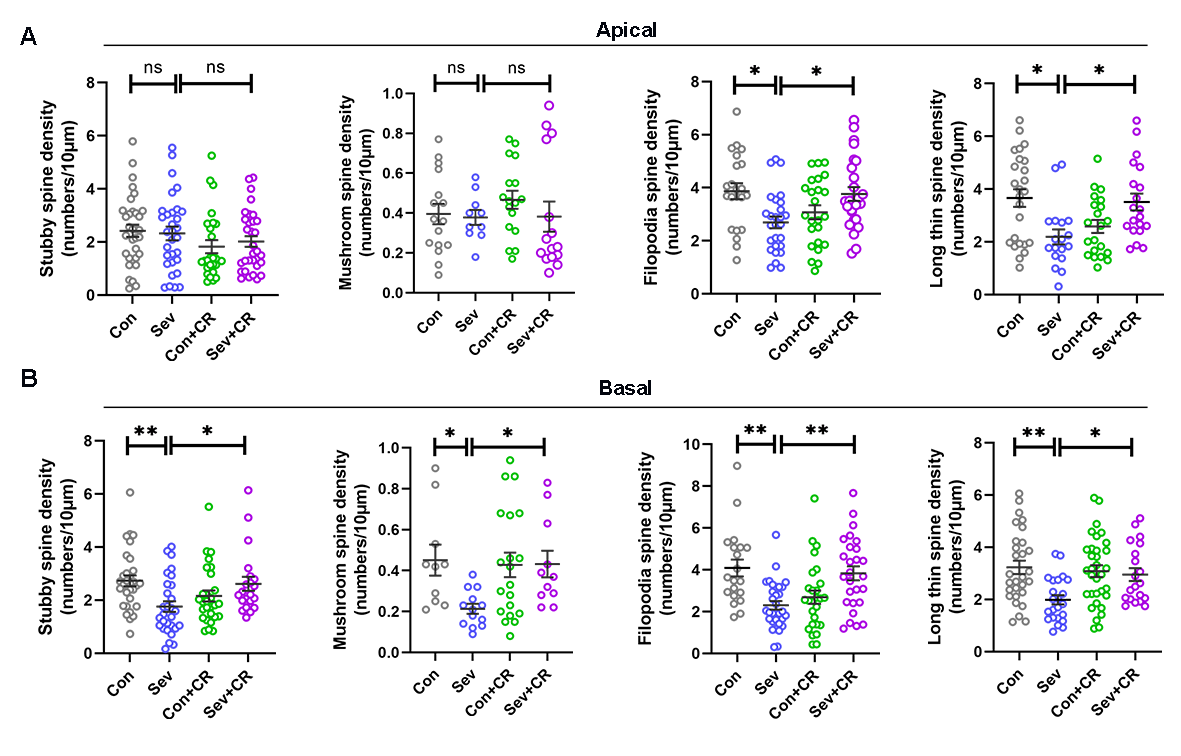

Supplement: Supplementary 1 — Figs. S1 to S9 [file research.0482.f1.zip › Supplementary Figure 5.tif]

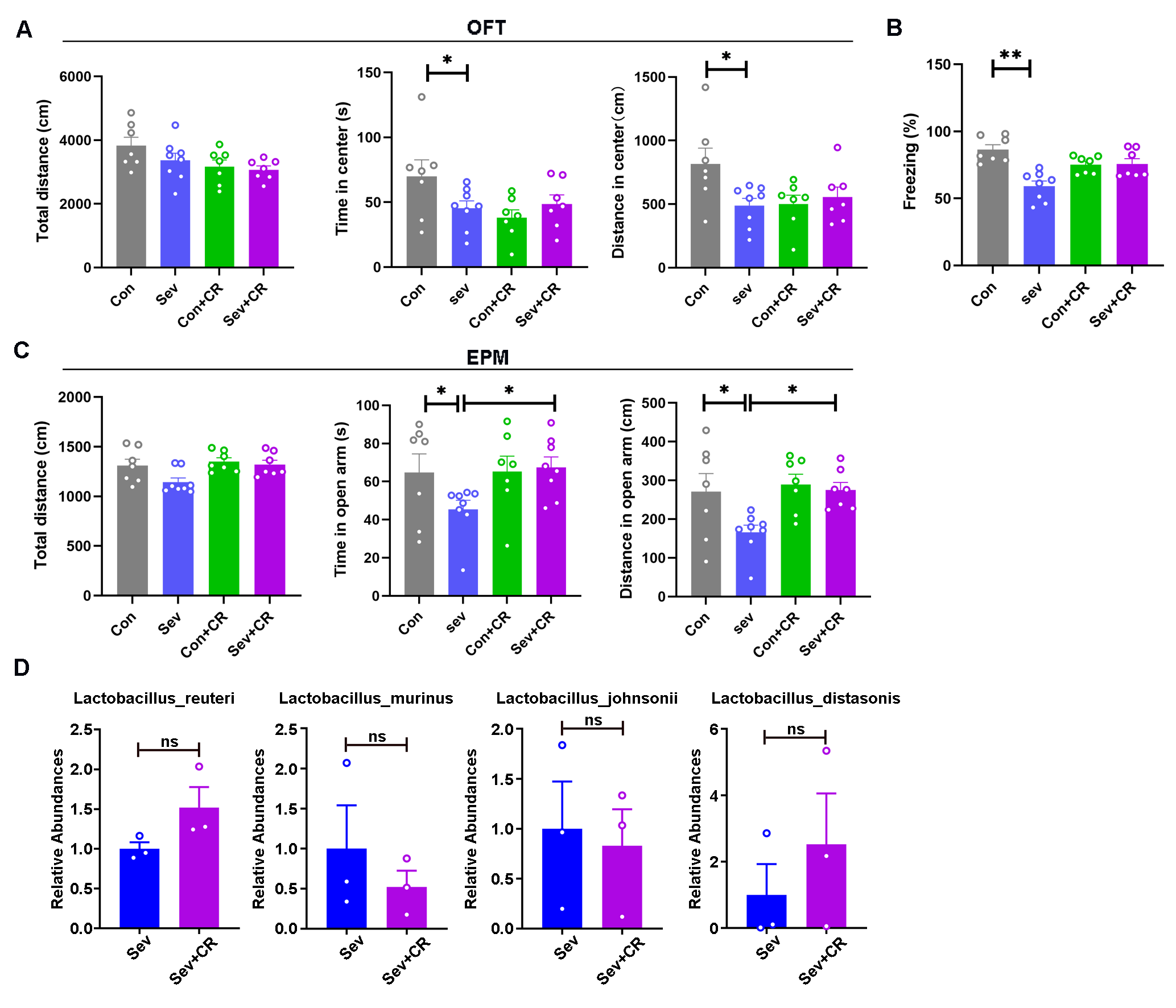

Supplement: Supplementary 1 — Figs. S1 to S9 [file research.0482.f1.zip › Supplementary Figure 6.tif]

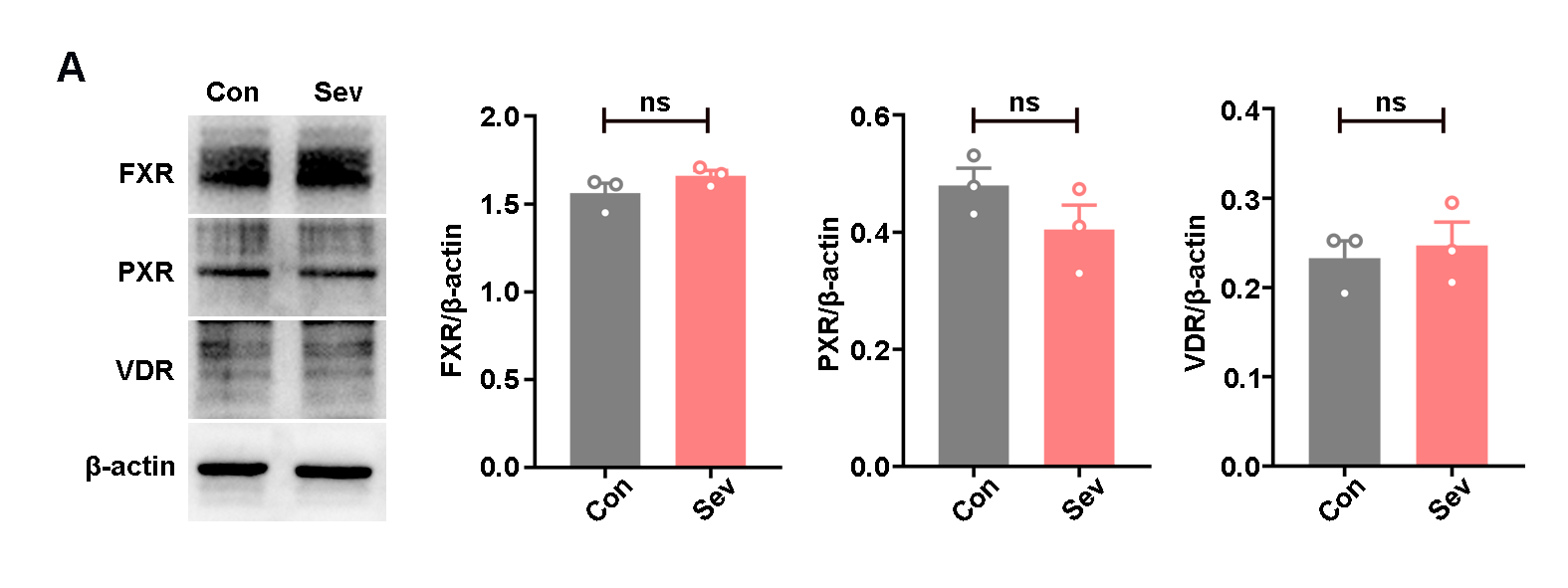

Supplement: Supplementary 1 — Figs. S1 to S9 [file research.0482.f1.zip › Supplementary Figure 7.tif]

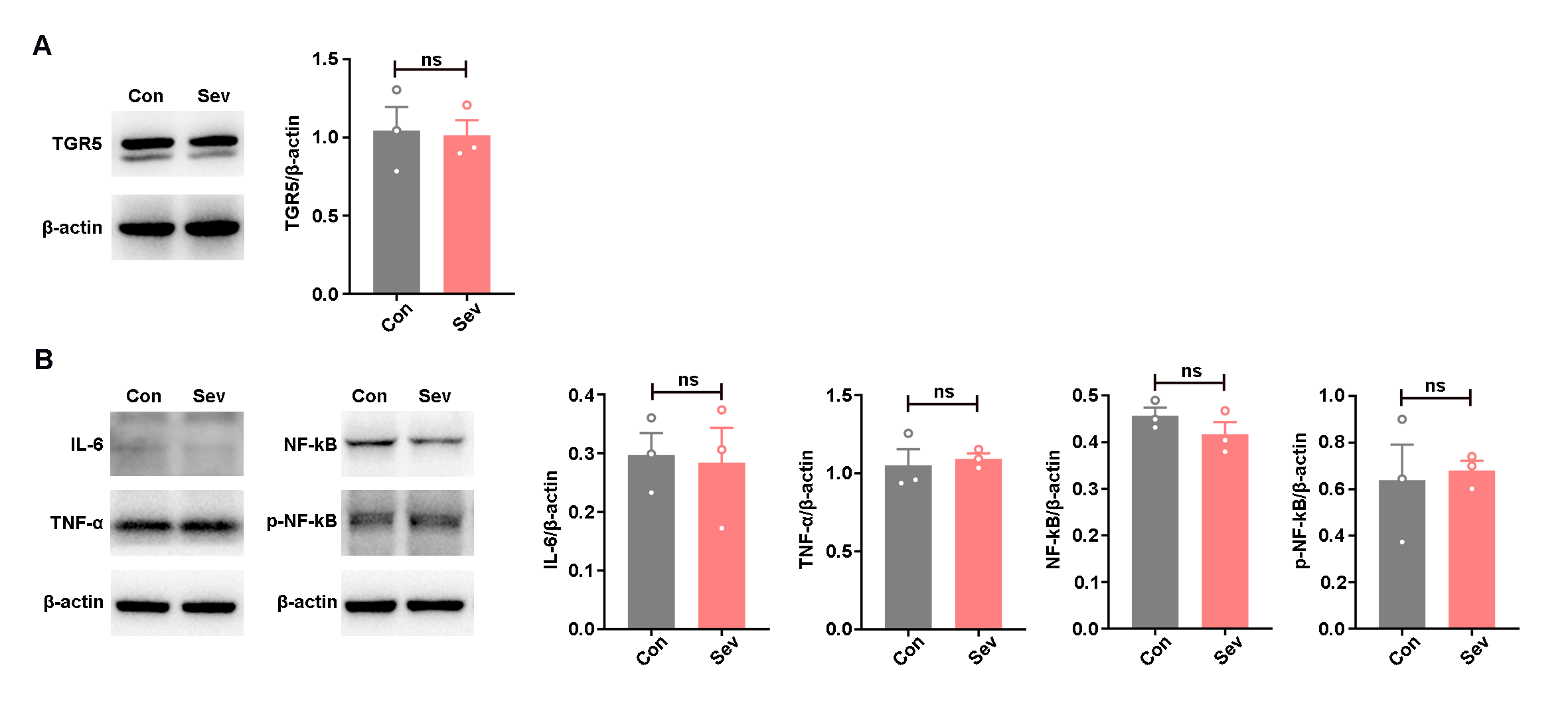

Supplement: Supplementary 1 — Figs. S1 to S9 [file research.0482.f1.zip › Supplementary Figure 8.tif]

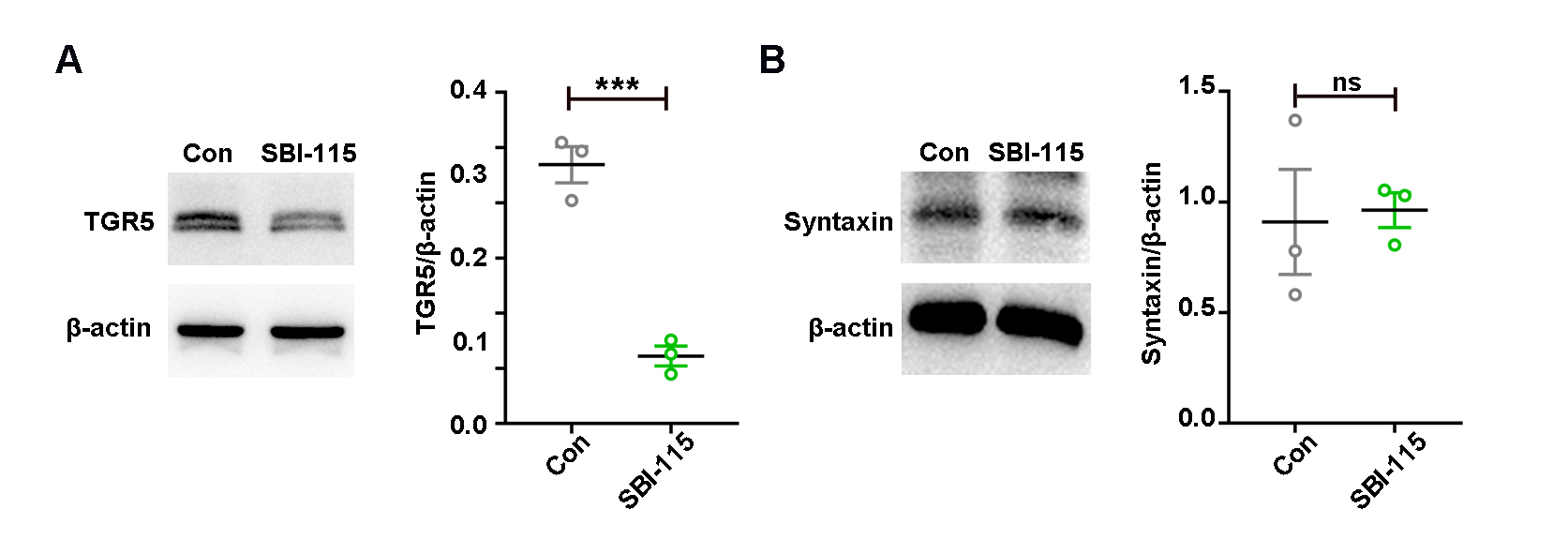

Supplement: Supplementary 1 — Figs. S1 to S9 [file research.0482.f1.zip › Supplementary Figure 9.tif]
